# Ethical considerations for the use of brain–computer interfaces for cognitive enhancement

**DOI:** 10.1371/journal.pbio.3002899

**Published:** 2024-10-28

**Authors:** Emma C. Gordon, Anil K. Seth

**Affiliations:** 1 Department of Philosophy, University of Glasgow, Glasgow, United Kingdom; 2 Sussex Centre for Consciousness Science and Dept of Informatics, University of Sussex, Brighton, United Kingdom; 3 Program on Brain, Mind, and Consciousness, Canadian Institute for Advanced Research, Toronto, Canada

## Abstract

Brain–computer interfaces (BCIs) enable direct communication between the brain and external computers, allowing processing of brain activity and the ability to control external devices. While often used for medical purposes, BCIs may also hold great promise for nonmedical purposes to unlock human neurocognitive potential. In this Essay, we discuss the prospects and challenges of using BCIs for cognitive enhancement, focusing specifically on invasive enhancement BCIs (eBCIs). We discuss the ethical, legal, and scientific implications of eBCIs, including issues related to privacy, autonomy, inequality, and the broader societal impact of cognitive enhancement technologies. We conclude that the development of eBCIs raises challenges far beyond practical pros and cons, prompting fundamental questions regarding the nature of conscious selfhood and about who—and what—we are, and ought, to be.

## Abstract 2

The use of invasive brain-computer interfaces holds great potential for not only medical applications, but also improving human cognition. This Essay explores the ethical, scientific and practical challenges posed by the use of this technology for cognitive enhancement.

## Introduction

**Fig 1:**
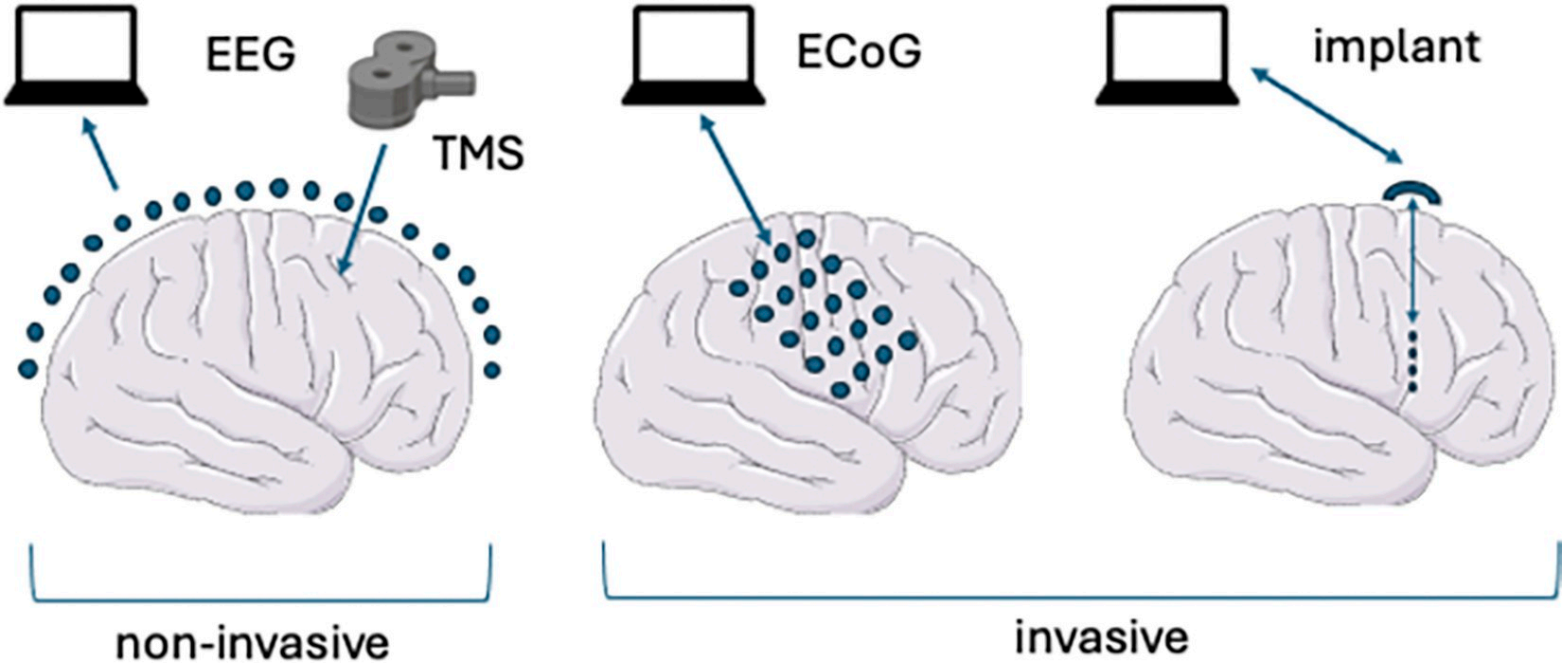
Varieties of BCI. Noninvasive BCIs record from outside the skull using methods such as EEG. These methods are usually used only to record brain activity, but can be combined with brain stimulation methods such as (noninvasive) TMS. Invasive BCI methods include ECoG in which grids of electrodes sit on top of the brain surface, underneath the skull, and electrodes implanted inside the brain. ECoG and implanted electrodes can both record and stimulate brain activity. Brain icon by Servier https://smart.servier.com/ is licensed under CC-BY 3.0 Unported https://creativecommons.org/licenses/by/3.0/. BCI, brain–computer interface; EEG, electroencephalography; ECoG, electrocorticography; TMS transcranial magnetic stimulation.

Brain–computer interface (BCI) technologies have been around for decades [1,2]. The underlying principles are simple: BCIs can be invasive or noninvasive (Fig 1). A typical invasive BCI consists of electrode probes inserted into the brain, which can record and/or stimulate neural activity in specific brain regions. The probes are connected to a computer (outside the brain and usually outside the body) that can process the recorded signals in various ways. These BCIs can record both local field potentials and—through application of spike sorting algorithms—spike trains from individual neurons. When BCIs are used in the “inside out” direction, they can control an external system such as a prosthetic limb or a speech synthesiser [3,4]. When they are used in an “outside in” direction, they can be used to drive neural activity to bring about changes in the brain, mind, and body. Bidirectional BCIs work in both directions, creating opportunities for closed-loop neurofeedback.

Another variety of invasive BCI is based on electrocorticographic (ECoG) recordings, which involves surgical implantation of a grid of electrodes that lie on the surface of the cortex, beneath the skull, but which do not penetrate the neural tissue [5]. ECoG grids provide wider coverage than implanted electrodes, but do not allow the identification of individual neurons. While the majority of ECoG-based BCIs are used in recording (inside out) mode, they can also be used for stimulation [6].

Noninvasive BCIs, as the name suggests, do not require surgical implantation. Instead, they rely on wearable technologies such as electroencephalogram (EEG) and functional near-infrared imaging [7]. Noninvasive BCIs are easier to deploy, but will generally lack the precision of invasive BCIs and will also generally lack the ability to stimulate brain activity, exceptions may include coupling noninvasive recording methods with noninvasive stimulation methods such as transcranial magnetic stimulation (TMS) and transcranial electrical stimulation. In this Essay, we focus on invasive BCIs.

Despite their long history [8], BCIs have recently gained prominence in the neurotechnological landscape. In part, this has to do with the entry of new companies such as Neuralink, which have strong public profiles. In addition, technological advances in electrode design, and in the possibilities afforded by recent advances in artificial intelligence (AI) and machine learning for analysing recorded neural data. There is now widespread agreement that BCIs could quite soon develop into a market worth billions of dollars annually [9]. However, two application domains for BCIs need distinguishing. These domains partially overlap in practice, but they have largely distinct aims. The first domain is medical or clinical treatment, and the second domain concerns neural and/or cognitive enhancement.

Clinical applications motivated the first BCIs, with the first human clinical trials dating from the 1990s [10], and they remain the dominant goal for most BCI developers. Inside-out BCIs have been used to help decode movement and speech commands from paralysed patients [11,12]. Outside-in BCIs have been used to ameliorate symptoms of Parkinson’s disease, control epilepsy, and relieve severe depression [13]. Some of these applications come under the rubric of “deep brain stimulation.” Many other applications—such as restoring vision in blind people, treating chronic pain, and bypassing damaged parts of the spinal cord—are at various stages of development [14]. This progress includes restoring abilities to communicate. To give a sense of the current state of progress in line with BCI development in this case, in just the two year span between 2021 and 2023, paralysed patients have gone from being able to use BCIs to communicate at 15 words per minute to 78 words per minute, achieved by a volunteer collaborating with a team at the University of California San Francisco [15]. This and other therapeutic applications of BCIs evidently promise many benefits, though as with any medical intervention there are ethical issues to consider, including risk/benefit ratios, data privacy issues, and patient dependence on continued existence of the BCI company for support and maintenance.

In this Essay, we focus on the second application domain: BCIs for neurocognitive enhancement in people who are not (necessarily) suffering from any specific BCI-related medical condition [16]. Cognitive enhancements improve our cognitive functionality in ways that go beyond correcting pathology, making us “better than well” in some way. Such applications may involve both the enhancement of existing capacities (e.g., memory and attention) as well as the potential creation of new capacities [17,18].

Although BCIs for enhancement are not generally immediate goals for BCI developers, prominent actors such as Neuralink and Synchron explicitly highlight this possibility as a medium- to long-term objective. For example, Neuralink’s mission statement is to “restore autonomy to those with unmet medical needs today, and to unlock human potential tomorrow.” The cyborg future envisaged by Neuralink and others raises ethical issues far more complex than those raised by current therapeutic medical applications (see, e.g., [19]). Here, we consider a range of these issues, as well as some potential responses that we contextualise within wider debates about the boundaries of the mind. We start by setting out some constraints on the development of enhancement BCIs (eBCIs), as well as the potential they hold. We then examine a range of ethical issues attending eBCIs, including privacy, inequality, standardisation of thought, inauthenticity, and cheapened achievements Altogether, we hope to provide a perspective on eBCIs that cautiously anticipates ways that embracing them would potentially improve, but perhaps also worsen, our lives.

## Current potential and limitations of BCIs

Although the basic principles of all BCIs are straightforward, and although recent progress has been impressive, there are many constraints that will likely limit and shape the trajectory of this technology, especially regarding uptake for enhancement purposes. These constraints can be divided into two (overlapping) categories: engineering and scientific.

All forms of BCI face many engineering challenges. Current invasive BCI probes record from only a tiny fraction of neurons. The current state of the art lies at around 1,000 electrodes, each typically detecting only a few individual neurons after software-based spike sorting. This is a tiny sample of the total cortical neuronal population (about 16 billion in adult humans). Current ECoG grids are also limited to a few hundred surface electrodes [15]. Advances in engineering and materials science are promising major improvements in electrode number and density, potentially reaching many thousands for both depth electrode and ECoG approaches [20]. For depth electrodes, there is also the potential to implant multiple locations, widening coverage [21]. But even with these advances, only a tiny fraction of the brain’s neurons will be sampled.

Some other engineering challenges pertain specifically to implanted electrodes, which have a tendency to detach, and to move around after implantation, potentially damaging surrounding neural tissue. Preventing inflammation and other damaging immune responses to BCI implantation is also difficult, as is maintaining long-term power and functionality. Again, new approaches may alleviate some of these difficulties, for instance through the development of highly flexible probes and conformal ECoG grids [20].

Scientifically, the challenges are even greater. It barely needs saying that understanding how the brain works is still a scientific project in relative infancy. But doing so does serve to draw a contrast with other human accomplishments, such as space flight and particle accelerator design. These accomplishments posed enormous engineering challenges, but were based on solid, well understood, and sufficient scientific foundations.

It seems very likely that the enhancement potential of BCIs will depend on a deeper understanding of how to read from, and stimulate, the relevant parts of (and patterns of activity within) the brain. This knowledge will itself depend on a deeper understanding of the fiendishly complex neural circuitry involved in perceptual, cognitive, and motor processes. This constraint goes hand-in-hand with the engineering constraints having to do with limited access to this neural circuitry. It has become increasingly apparent that neurocognitive processes are difficult to localise to a single brain region or neuronal assembly. Also, most or all such processes involve not just neuronal activity, but encompass a broad range of neurophysiological factors, including chemical neurotransmitters and activity of non-neural brain cells such as glial cells. In the other direction, an influential literature in the philosophy of mind and cognitive science—the “extended mind”—emphasises that cognitive abilities extend beyond the brain to include the body and parts of the external environment, including tools such as notebooks and mobile phones [22–24]. This means that there is unlikely to be any simple correspondence linking a desired enhancement goal (e.g., improved memory) and modulation of some single neuronal target (e.g., neurons in the hippocampus).

Some mitigating factors are worth noting. First, BCIs themselves may help address the scientific challenges, by allowing greater precision when recording and stimulating neural populations, and through testing of specific causal hypotheses using neurofeedback. Some BCI technologies, such as those leveraging optogenetics in nonhuman animals, are particularly promising in this regard, but—thanks to their technical constraints—are currently out of range for human applications [25].

Second, perhaps the scientific challenges can be to some extent evaded. In the outside-in direction, machine learning algorithms can allow powerful classification and prediction given sufficient data without any (or very much) understanding of the processes that generated these data. (Sufficient data, in general, means a very large amount of data indeed.) In the outside-in direction, the human brain seems to be remarkably adaptable to the relatively uniformed and unnaturally structured inputs that current BCIs make use of. To some extent—especially for perceptual systems—the brain seems to be “plug and play” [26].

These mitigating factors remain just that. They may soften but they do not get close to eliminating the relevant scientific challenges in understanding precisely how, where, and when to intervene in, and record from, specific neuronal populations in order to enhance existing cognitive capabilities or install new ones. This is why near-term eBCI applications may be limited to controlling apps on phones or other similarly prosaic activities. This may well be game-changing for people with medical conditions such as paralysis, but wider uptake may be limited.

In spite of these considerable challenges, BCIs still hold promise for enhancement applications. To better understand what might be possible, we now turn to the enhancement potential of BCIs, exploring how they could in principle, and with further developments, augment human capabilities.

## Enhancement potential of BCIs

How might eBCIs, aimed not at alleviating neurological disease or pathology, but rather human enhancement, “unlock human potential”? While this question occupies its own emerging research area in bioethics [19], a useful framing for this question comes from the “extended mind” setting introduced earlier [23]. eBCIs provide many new ways in which extended cognitive processes can be implemented. They offer an immediacy and directness that seems—at least in principle—substantially different from that offered by other extended mind situations, such as those involving notebooks and phones.

The enhancement potential of eBCIs applies both to individuals, and to society at large. Consider what may be possible if—for the moment—we set practicality and feasibility to one side and give imagination free reign. One can imagine eBCIs that enhance memory, by providing instant thought-based access to both autobiographical and semantic information stores. eBCIs might improve attentional focus by directly modulating neural circuits involved in sustaining attention (see [27] for a clinical example in children with ADHD). Relatedly, eBCIs might also be able to modulate mood, in order to allow people to function more effectively in various demanding situations [28,29]. Perhaps eBCIs could allow people a wider bandwidth of control and communication by transcending the limits of having only 1 mouth and 2 hands. More generally, eBCIs might bring about substantial epistemic benefits, by dramatically changing capacities for knowledge access and discovery. Language barriers might become a thing of the past, once eBCIs can implement direct thought-based translation. Even more provocatively, could eBCIs increase the “bit rate” of human cognition, as the Neuralink founder has suggested [30]?

Other examples arise when we widen the lens. eBCIs might be able enhance our physical prowess as well as our cognitive capability, perhaps by improving reaction times or sensory sensitivity. And why not use eBCIs to endow us with entirely new sensory and perceptual modalities, by stimulating the brain with signals derived from things like sonar, compass direction, or even current stock prices [31]? Consider the potential enhancement benefit of being able, as technology advances (though for challenges here see [32]), to control additional robotic limbs, when two hands are not enough. Or consider, for example, being able to decode a stream of thought about one topic, while simultaneously holding a conversation about another. What about being able to dramatically accelerate your rate of thinking, much as you might speed up the playback rate for an audiobook?

Then there are wider benefits that may accrue to society at large. If many people augment their individual cognitive prowess, perhaps this could lead to more efficient and effective coordination and synthesis of information, leading in turn to enhanced capability to address societal challenges such as curing disease, mitigating climate change, and the like. eBCIs might even allow direct brain-to-brain interactions, so that the extended mind now becomes an extended collective mind, with additional capabilities that would be difficult to anticipate (see [33]). However, these more ambitious applications have their own unique challenges. Such scenarios are not just implausible by the scientific and engineering criteria discussed earlier; they are psychologically and philosophically implausible too.

We have already noted that the neurocognitive architecture of a human being is deeply intertwined with its embodied and embedded existence. Whereas implementing a novel perceptual modality may be relatively “plug and play,” implementing the ability to hold two simultaneous conversations, for example, would likely require a wholesale reorganisation of our entire neurocognitive architecture, and likely the body as well. And the pacing of cognitive activity—our “speed of thought”—may have difficult-to-overcome upper bounds, no matter what the bandwidth limitations are imposed on brain–body–world interactions [34]. What’s more, embodied aspects of cognition are not necessarily limitations but can also usefully considered as part of the generative process itself. How do I know what I think until I see what I say?

These feasibility constraints substantially undermine the likelihood of a future in which we all pop along to our high-street neurosurgeon, install the latest eBCI, and emerge newly superintelligent—and with a new hole in the head. It is true that if one allows imagination free reign, there seems to be little limit on how eBCIs could transform our lives and our society. And if our imagination were also enhanced by eBCIs, then what is unimaginable now might become imaginable in the future. But this kind of untrammelled neuro-utopianism is useful only to sketch the space of possible futures. Our imagination ought not be given entirely free reign, but—to be useful—should be reined in by what is, and what is likely to be, practical, feasible, and ethical. With this in mind, we now turn to the ethical considerations.

## Ethical concerns raised by eBCIs

We have now unpacked some of the main ways in which eBCIs have the potential to enhance us, and we have highlighted various hurdles—engineering, scientific, and psychological. The present section takes us into philosophical territory: on the assumption that we can create eBCIs that satisfactorily address practicality/feasibly issues, should we? Do any considerations count against a “full steam ahead” approach to eBCI development, if this development became practically feasible? In this section, we canvass six key philosophical challenges that any affirmative answers will need to grapple with (see Box 1). In addressing these challenges, we do not give imagination entirely free reign, but instead focus on what might be possible given conceivable practical advances. We do this because the ethical issues can be made more vivid by focusing on eBCIs more advanced than are presently feasible. That said, the issues we identify are all at least in principle applicable to all eBCIs.

**Box 1: Key ethical questions surrounding invasive BCIs for cognitive enhancement**

Erosion of privacy: To what extent does widespread use of eBCIs raise the risk of privacy erosion, such as thought violation or “brainjacking” where bad actors gain illicit access to neural data?

Inequality: Could giving financially privileged individuals access to eBCIs further widen existing socioeconomic gaps by providing them an additional cognitive or intellectual advantage?

Mental monoculture: Could the widespread use of eBCIs standardise cognitive processes, leading to diminished cognitive diversity and hindering innovation?

Inauthenticity: Do eBCIs pose a threat to authenticity by altering cognitive functions in ways that conflict with living in alignment with one’s true or evolving self?

Cheapened achievements. Are achievements aided by eBCIs, like cognition-boosting devices, less valuable due to reduced effort, with reliance on enhancements shifting credit from the individual to the tool’s creator?

Loss of autonomy vs hyperagency: Do eBCIs pose a “dual challenge” by potentially undermining autonomy through misinterpreted commands, while also creating “hyperagency,” where enhanced capabilities increase personal responsibility and the pressure of higher ethical standards?

### Erosion of privacy

The idea that we have a right to freedom over our thoughts has been traditionally discussed in connection with freedom to express our thoughts in action (e.g., protest and speech). What about the thoughts themselves? Various international law frameworks recognise a right not to have one’s thoughts or opinions revealed [35] or manipulated [36]. The introduction of eBCIs in a population raises the risk of this kind of thought violation.

Consider, for instance, the threat of “brainjacking”—where bad actors attempt to gain unauthorised (at least, indirect) access to what you are thinking by gaining access to neural data and data pertaining to which thought commands are implemented by the eBCI, or to your neural data more generally [37–40]. Such data could evidently be used for all kinds of nefarious purposes: knowledge of your emotional state could be used to exploit marketing opportunities, or—more dramatically—knowledge of intentions could lead to blackmail. A variation of these risks arises when we consider that interpretation of eBCI data is likely less than fully accurate. On the one hand, this implicitly restores some vestige of privacy, but on the other it opens new vulnerabilities; for example, we might lose access to finance on the basis of a misread intention to gamble wildly.

The possession of such information also raises risks of various kinds of thought manipulation. For example, the more information about one’s mental activity and patterns is available to third parties, the more likely (as one concern goes) that content and timing of stimuli might be optimised for persuasive impact [41,42]. This remains within the inside-out (reading) direction. Some deeper concerns arise when considering the outside-in direction. Early BCI experiments, dating back to seminal work of Wilder Penfield in the 1930s, showed that targeted brain stimulation could elicit a desire to move [43]. More fine-grained versions of this technique raise the possibility of precision implantation of specific intentions—changing not only what somebody does, but what they wish to do. As Arthur Schopenhauer [44] put it: “man can do what he wills, but he cannot will what he wills.” eBCIs raise the prospect of third-party actors changing what a person “wills,” perhaps without their consent [45].

A related worry has to do with ownership of neural data [46]. Already there is legal uproar about ownership and fair use of the text and images scraped from the internet to train the generative AI models underlying text and image production. The widespread deployment of eBCIs could easily lead to similar challenges arising for individuals’ neural data. As neurotechnologist Grégoire Courtine put it: “we do intend eventually to have all this brain information in the cloud, so we can train a large language model and create a brain GPT. Then we can learn from hours and hours of brain activity from a lot of people” [47]. Guidance exists for medical BCIs (for example, in the USA FDA, and also in the EU—see [48]); but presently consumer BCIs lack the same kind of regulation, although some progress is being made (see Box 2) [19].

**Box 2: Current initiatives and recommendations for eBCIs**

Several efforts are already underway to define a the landscape for eBCI development and regulation. For example, recommendations that privacy of thought ought to be enshrined, protecting individuals’ rights to cognitive liberty and establishing safeguards against unauthorised access to neural data. In the USA, for example, two states (Colorado and Minnesota) have specific regulatory guidance for eBCIs for consumer purposes. One step forward here is Colorado’s recent inclusion of neurological data in the Colorado Privacy Act. Minnesota has gone a step further proposing legislation that supports civil and also criminal penalties for violations of neural data rights in cases of consumer BCIs; this has, as of May 2024, been signed into law by the Minnesota governor Tim Walz. International non-legally binding recommendations include the OECD’s Recommendation on Responsible Innovation in Neurotechnology (2019) and their more recent iteration (2023), as well as UNESCO’s 2023 Declaration on the Ethics of Neuroscience and Neurotechnology.

Recommendations are also advising that extensive cognitive testing should be conducted to characterise psychological consequences of eBCIs, both narrow (e.g., mood manipulation, vulnerability to influence) and broad (e.g., change of personality, loss/transfer of agency). Finally, it is argued that eBCIs should be regulated as if they are medical devices, i.e., applying stringent safety and efficacy standards and ensuring proper medical oversight and quality control. These recommendations are all good ideas, though their implementation may of course face practical hurdles.

### Inequality

Suppose eBCIs are available in the open marketplace; either they are available to everyone cheaply, or they are not. Let us assume they are not, and that like other high-end tech products they are expensive and will likely remain so. In this scenario, eBCIs would be of limited access, available only to the most privileged—thereby giving those already most financially privileged an additional cognitive or intellectual advantage, potentially widening already existing socioeconomic gaps [49–51]. Let us now take this worry a bit further; if the wealthy have cognitive advantages that lead to more wealth and further cognitive advantages (in a reinforcing feedback loop), there is a downstream concern that we might face a deeper societal “cognitive divide” between enhanced and unenhanced individuals, where the abilities of one group are increasingly distinguished from the abilities of the other.A common response to concerns like these is that benefits initially restricted to financially privileged people will over time become available to wider segments of society as the relevant technology develops and becomes more efficiently delivered. Mobile phones provide one good example of this effect, among many that could be given. However, this kind of “trickle down” process does not always apply; not everyone has a laptop computer, and the rationale itself has been widely criticised in its more general economic instantiation [52]. Also, in some contexts—including potentially eBCIs—the self-reinforcing dynamics may be such that the initially benefiting group bootstraps itself away from other groups, despite some aspects of the technology becoming more widely available, leading to the permanent societal cognitive divisions mentioned above.

### Mental monoculture

Let us now suppose that eBCIs do indeed become available easily and cheaply for all, at least in some form. Here is a very different challenge, predicated on equality rather than inequality of access: if (in short) everyone has an eBCI, a risk that arises concerns uniformity of thought and the implications for culture (scientific, artistic, etc.) and wider society that follow from that. Here is one way we can envision this challenge unfolding. Suppose eBCIs lead to a standardization of certain kinds of cognitive processes as users adapt to the interface’s parameters, or (relatedly) the algorithms that eBCIs use might turn out to favour certain thought patterns or be optimised for certain languages or cultural contexts. This might initially happen because of the nature of the training data used to optimise the eBCI, which is unlikely to be representative of the full range of cognitive diversity in society. As users become used to thinking in a particular way in order to effectively utilise eBCI technology (and they may well be forced to do so, in order to not fall behind economically—see the previous point), then these patterns of thinking may become habitual and present more pervasively in users’ mental lives. The upshot would be less cognitive diversity over time—and at worst a kind of mental monoculture.

Would this be a bad outcome? Diminished cognitive diversity might indeed be bad news when it comes to progress. For example, there is evidence that cognitively diverse teams are better at problem solving than cognitively nondiverse teams [53]. Cognitive diversity is also important for innovation [54], including scientific and artistic creativity [55]. There is also the wider worry that a mental monoculture would lead to societal stasis as the costs of thinking in new ways increase. This could happen even with well-intentioned eBCI development. With malign intentions, one can imagine the imposition of a mental monoculture specifically to serve the interests of perpetuating power dynamics.

Note a potential caveat: language users are capable of learning new languages without losing the ability to speak their original language. Perhaps this will also happen with eBCIs; we may learn to think in a particular way by using the eBCI, but this will not interfere with our non-BCI ways of thinking. This could be the case—but it seems unwise to assume it without detailed study. This is particularly so given a potentially relevant disanalogy: language users who do learn a language often do this purposively, aware that they are embracing a new way of representing the world linguistically. In the eBCI case, the acquisition of new ways of thinking imposed by the eBCI might be more subtle, such that one might be less appreciative of the ways their thinking is changing than they will (typically at least) be when intentionally taking themselves to be embracing new linguistic capabilities.

In these ways, whereas inequality of access to eBCIs seems to risk a cognitive divide, equality of access risks cognitive homogeneity. Note that these worries can coexist and may even reinforce each other. It could be that especially powerful eBCIs remain of limited access, increasing cognitive stratification, while widespread availability of less powerful eBCIs still risks homogeneity. It could even be that social or financial privilege may allow access to “bespoke” eBCIs which, in virtue of being more sophisticated (and expensive) do not impose the same requirements on “how to think” that future mass-market eBCIs would do, in order to remain viably mass-market.

### Inauthenticity

Bioconservative philosophers have long argued that enhancements, including eBCIs, might compromise our authenticity. The core idea is that living in accordance with one’s “true self” is a crucial component of human flourishing [56] and using eBCIs to alter our cognitive function may conflict with being true to ourselves.

The extent to which we should be concerned about eBCIs affecting our authenticity largely depends on our understanding of the “true self” [57]. For example, essentialist views [58] propose that the true self is made up of some set of consistent, core traits. On these views, we can gain a deeper understanding of our true self through self-exploration, but to live authentically, we must keep our “natural” form as unaltered as possible. If this view is correct, it poses a challenge for eBCI advocates, as avoiding these devices would be necessary for maintaining authenticity.

However, eBCIs may be less threatening to authenticity when paired with an existentialist approach to authenticity (e.g., [59–61]), which says that that being true to oneself means living in accordance with values that one has reflectively endorsed. From this perspective, if using an eBCI aligns with an individual’s endorsed values, there is no apparent reason to believe that one is less authentic when an eBCI is installed [62].

In the above approaches there is a stable self that has values that are either unchanging (essentialist) or potentially malleable under reflection (existentialist). Another possibility, and one increasingly emphasised in philosophy of mind and consciousness science, is that there is no single stable self. That is, the experience of “being a self” is a kind of perceptual and cognitive construction, composed of many different aspects, and open to change on all or most [63–65]. Sometimes, changes in experienced selfhood can be abrupt and dramatic, perhaps following a brain injury or disease, as in the notable cases of Phineas Gage and Clive Wearing [66,67]. But even without dramatic events, it is plausible that one’s experience of being a particular self is always changing. And because these changes are relatively gradual, there is no reason to expect that any of us would experience the change itself. A long tradition of experiments in “change blindness” reveal that change of experience need not always entail a corresponding experience of change [68].

On the one hand, recognising the instability of the self may ameliorate authenticity concerns, because it opens the possibility of remaining dynamically “true” to a self that is always changing. On the other hand, these concerns are arguably exacerbated because eBCIs might accelerate changes in experienced selfhood; if one’s self has changed in ways that would not have happened without an eBCI implant (and perhaps in ways that depend on the motivations of the BCI provider), is this not a challenge to authenticity? There are complex issues hereabouts: for example, how should we think about an eBCI-induced change in self-experience if that change were anticipated and desired by the eBCI-user? An example here might be someone who decides to install an eBCI in order to become the kind of person who no longer has an addictive personality. This might seem benign, but now consider the situation if the desire to change one’s self itself derived from the use of the eBCI. This is much trickier situation, where much depends on how and why the eBCI led to the emergence of the desire for self-change.

### Cheapened achievements

Imagine there are two mathematicians who independently develop a successful new proof. One comes up with the proof through the assistance of a cognition-boosting eBCI while the other does not rely on one, opting instead for a more traditional approach. Does the former have a less valuable achievement on account of having relied on the eBCI? Some bioconservative thinkers say the answer is “yes” (whereas techno-optimists as well as more cautious bioliberals are sceptical [18]). One line of argument here [69] maintains that cognitive enhancements like eBCIs divorce performance from the kind of effort needed to make the achievement valuable, or at least as valuable as it would be otherwise absent the eBCI. Whether, and if so why, effort makes achievements valuable remains contentious. According to Bradford [70], the explanation is that effort is related to valuable exertion of the will. This explanation becomes complicated if eBCIs change what people experience as “will,” as well as their ability to exercise it. In a similar vein, Sandel [71] argues that the more powerful the enhancement used, the more our admiration for what is accomplished shifts from the agent using the enhancement to the enhancement’s creator or developer.

Recent literature and bioethics suggests that the “cheapened achievements” worry is overstated as a general argument [18,72]; whether it has any teeth in the case of BCIs will plausibly depend on factors including the extent to which users rely on the eBCI as a mere tool as opposed to whether it is deeply integrated into our cognitive architecture [73] and whether eBCI users are exhibiting various other abilities and skills in the service of using eBCIs in effective ways [74].

#### Loss of autonomy versus hyperagency

A further philosophical challenge for eBCIs concerns autonomy, and in connection with “hyperagency” [71,75]. Suppose, to use a simple example, an individual issues a thought command to a computer via an eBCI; in the ideal case, this command will be executed by the eBCI exactly as the individual intends. We can envision, however, mismatch cases. In particularly bad cases (either through malicious third-party actors or simply through imperfection in design), suppose the eBCI in some way “overrides” or otherwise fails to reliably encode or implement the thought command (in some cases without the user realising); these cases threaten user autonomy by undermining (in potentially undetectable ways) their capacity to make free choices in line with her intentions.

But there is another side to this coin; imagine that eBCIs were highly effective and functioning in reliable alignment with our intentions, so much so that, equipped with the eBCI, we dramatically increase our power to fulfil our aims. Might such enhanced power come with a kind of “explosion” of responsibility that we are not yet ready for? Such a concern has been expressed by Sandel. As he sees it, when we become more capable via enhancement, we thereby become to that extent more responsible for how our lives go. Such a boost in personal responsibility, as he sees it, may have its own risks for our overall well-being; the more we are responsible for—i.e., the more we transform into “hyperagents”—the fewer excuses we have for mistakes or bad choices, and the standard to which we are held may be dauntingly high. Whether or not considerations about hyperagency represent an apt worry (see [75] for criticism), it invites reflection on how our ethical obligations might be increased on the presumption that our capacities are (through eBCIs) significantly greater.

## Conclusions and future perspectives

The balance between benefit and risk in eBCIs suggests the need for a middle way, somewhere between accelerationist/techno-optimist unrestricted development and outlawing nonmedical applications entirely. There is also the pragmatic issue that separating medical from enhancement applications will likely be very difficult in practice. Relatedly, the more obviously justifiable benefits of medical applications may put pressure on any attempt to introduce any form of regulation for eBCIs; people being offered relief from paralysis may quite understandably think that pontificating about potential mental monocultures to be irrelevant and obstructive. But what might this middle ground between techno-optimism and bioconservatism look like? A full discussion on this point is beyond the scope of what we can do here. However, it is worth emphasising that some efforts to define this landscape are already underway (Box 2).

In this Essay, we have focused on invasive BCI technologies. While potentially transformational, these technologies are less likely to see near-term widespread deployment than wearable BCIs based on EEG and other noninvasive imaging methods. There is already a vibrant consumer market for wearable BCI systems. As noted earlier, these technologies are typically limited to recording (inside-out) and have lower precision than invasive methods. Nonetheless, some of the ethical concerns we have raised—including erosion of privacy, uniformity of thought, cheapened achievements, and loss/gain of autonomy—apply to noninvasive eBCIs too. The noninvasive combination of ease of access, but lower precision and emphasis on recording, means that the overall ethical picture will look rather different [38].

One further issue to raise in closing is the apparent significance of the boundary of the skull. This boundary is crossed by all BCIs, though for invasive systems it is crossed in a more profound and permanent way. It is the crossing of this boundary that seems to be the most distinguishing features of BCIs—whether for enhancement or otherwise. After all, modern (non-BCI) technologies have led to all kinds of vulnerabilities in both of the directions we have identified: privacy (inside-out) and manipulation (outside-in). Social media, for example, both reveals our personal data and exposes us to manipulative pressures. BCIs take these same concerns and import them more directly into the brain. But why is this boundary significant? It does seem to have a prima facie lay significance. People may think that this boundary is important in the same way they might think that the integrity of one’s genome is important. A certain degree of squeamishness seems undeniable. But is there anything more substantive to say?

One possible line of thinking is that the boundary of the skull makes a difference because, broadly, it demarcates what we in principle have agency over. Recalling Schopenhauer [44], we can decide what to do, or say, but we cannot decide what to think. However, this line of thinking rapidly runs aground on the rocks of the debate over free will, and of the plentiful empirical evidence that we have less control over many over our actions than we might think [76,77]. The boundary is eroded in both directions. In the case of free will, the idea that the skull marks a meaningful divide behind which some sacrosanct conscious agent orchestrates behaviour is extremely difficult to maintain in the face of philosophical, theoretical, and empirical objections [64,76–78]. In the case of action control, many apparently voluntary actions unfold without accompanying experiences of intention or agency, and so have more in common with reflexive action than one might immediately think [79].

Nonetheless, a case can be made for the importance of the skull. This is simply that once one breaches this boundary, no further boundaries remain. The importance of the skull may therefore lie in preserving the idea of autonomy, rather than in preserving anything particular about autonomy or agency itself.

What’s clear from this discussion, and from other discussions of issues surrounding eBCI development, is that the questions raised are not limited to concerns about positive and negative individual and societal consequences. The merging of our neurobiology with technology raises fundamental questions about who, and what, we are—and who and what we can, and ought, to be.

## Abbreviations

AI: artificial intelligence
BCI: brain–computer interface
eBCI: enhancement brain–computer interface
EEG: electroencephalography
ECoG: electrocorticography
TMS: transcranial magnetic stimulation
